# Diagnosis and Excision of Glomangioma of the Lower Extremity

**DOI:** 10.7759/cureus.80458

**Published:** 2025-03-12

**Authors:** Aaryan Patel, Ishan Deshmukh, Robert Jones, Venkatasai Jonna, Constantino G Lambroussis, Abbas Merchant

**Affiliations:** 1 Physical Medicine and Rehabilitation, Lake Erie College of Osteopathic Medicine, Erie, USA; 2 Vascular Surgery, Lake Erie College of Osteopathic Medicine, Elmira, USA; 3 General Surgery, Olean General Hospital, Olean, USA; 4 Pathology and Laboratory Medicine, Cooper Medical School of Rowan University, Camden, USA; 5 Osteopathic Medicine/Family Medicine, Lake Erie College of Osteopathic Medicine, Elmira, USA

**Keywords:** complete excision, excision of tumor, extradigital glomus tumor, glomangioma, glomus tumor rare locations

## Abstract

Glomangiomas are rare, benign vascular tumors originating from the specialized arteriovenous structure known as the glomus body. These tumors typically occur in subungual or digital locations, such as the fingers and toes. However, extradigital glomangiomas, which develop in other areas of the body, are much less common. When present in atypical sites, these lesions often mimic more prevalent subcutaneous abnormalities, such as sebaceous cysts, lipomas, or dermatofibromas, posing significant diagnostic challenges for clinicians. The nonspecific clinical features of extradigital glomangiomas, particularly the absence of hallmark symptoms like severe localized pain or hypersensitivity, can further complicate the diagnostic process. This case report describes a 67-year-old male who presented with a 1-cm soft, mobile, and asymptomatic lesion near the right knee, initially presumed to be a sebaceous cyst. Following unsuccessful drainage attempts, the lesion was surgically excised. Histopathological examination confirmed the diagnosis of a glomangioma, with glomus cells arranged around branching vascular channels and positive staining for smooth muscle actin and vimentin. Glomangiomas should be considered in the differential diagnosis of persistent subcutaneous masses in unusual locations. Greater recognition of these rare lesions can facilitate timely and appropriate management.

## Introduction

Glomangiomas, also known as glomus tumors, are rare neoplasms that account for approximately 1.6% of all soft tissue tumors [1]. These tumors originate from the glomus body, a specialized structure composed of smooth muscle cells that line the walls of arteriovenous structures involved in thermoregulation. While these tumors are classically associated with subungual or digital locations, extradigital occurrences are far less common and often underrecognized. When found in atypical sites, glomangiomas frequently present diagnostic challenges due to their nonspecific clinical features and their ability to mimic more common lesions, such as sebaceous cysts, lipomas, or other subcutaneous abnormalities. These extradigital glomangiomas can occur in various parts of the body, including the trunk, extremities, and even the head and neck region, although they are extremely rare in these atypical locations [2]. Their nonspecific presentation, often lacking the hallmark symptoms seen in digital glomangiomas, can lead to significant delays in accurate diagnosis and appropriate treatment. Increased awareness and recognition of the potential for extradigital glomangiomas among clinicians is essential to ensure timely diagnosis and management of these rare but clinically important lesions [3-5].

Intradermal and soft tissue glomangiomas occurring in the region of the knee are exceedingly rare, with only 36 cases of glomangiomas around the knee being described in recent meta-analysis [5]. Unlike their more typical counterparts, extradigital glomangiomas may lack the hallmark symptoms of severe localized pain or hypersensitivity, further complicating diagnosis [6-9]. Despite their benign nature, accurate identification and surgical excision are essential for definitive treatment and to prevent recurrence [10].

## Case presentation

A 67-year-old male patient presented with a 1-cm soft, mobile, and otherwise asymptomatic subcutaneous lesion located near his right knee. Based on the initial clinical examination and patient presentation, the lesion was suspected to be a sebaceous cyst, a common subcutaneous abnormality. However, the lesion demonstrated resistance to attempts at drainage, which did not resolve the lesion. This persistent nature and the failure of the lesion to resolve with conservative management necessitated the decision to proceed with surgical intervention and excision of the mass of the lesion for further evaluation and definitive diagnosis.

The procedure involved cleaning of the area with ChloraPrep and maintaining sterile technique. Local anesthesia was administered with 5 mL of 1% lidocaine with epinephrine. A 1.5-cm incision was made over the cyst, and the surgeon carefully dissected through the subcutaneous tissues to expose a 1-cm cyst-like structure. The mass was then removed using blunt and sharp dissection techniques. The surgical specimen was then sent for comprehensive pathological analysis. To close the incision, the surgeon used 3-0 Vicryl interrupted subcuticular sutures, and the wound was dressed in 4x4 gauze and Tegaderm. The patient tolerated the procedure well, with only minimal blood loss of approximately 2 mL. The specimen was promptly forwarded to the pathology laboratory for further evaluation.

**Figure 1:**
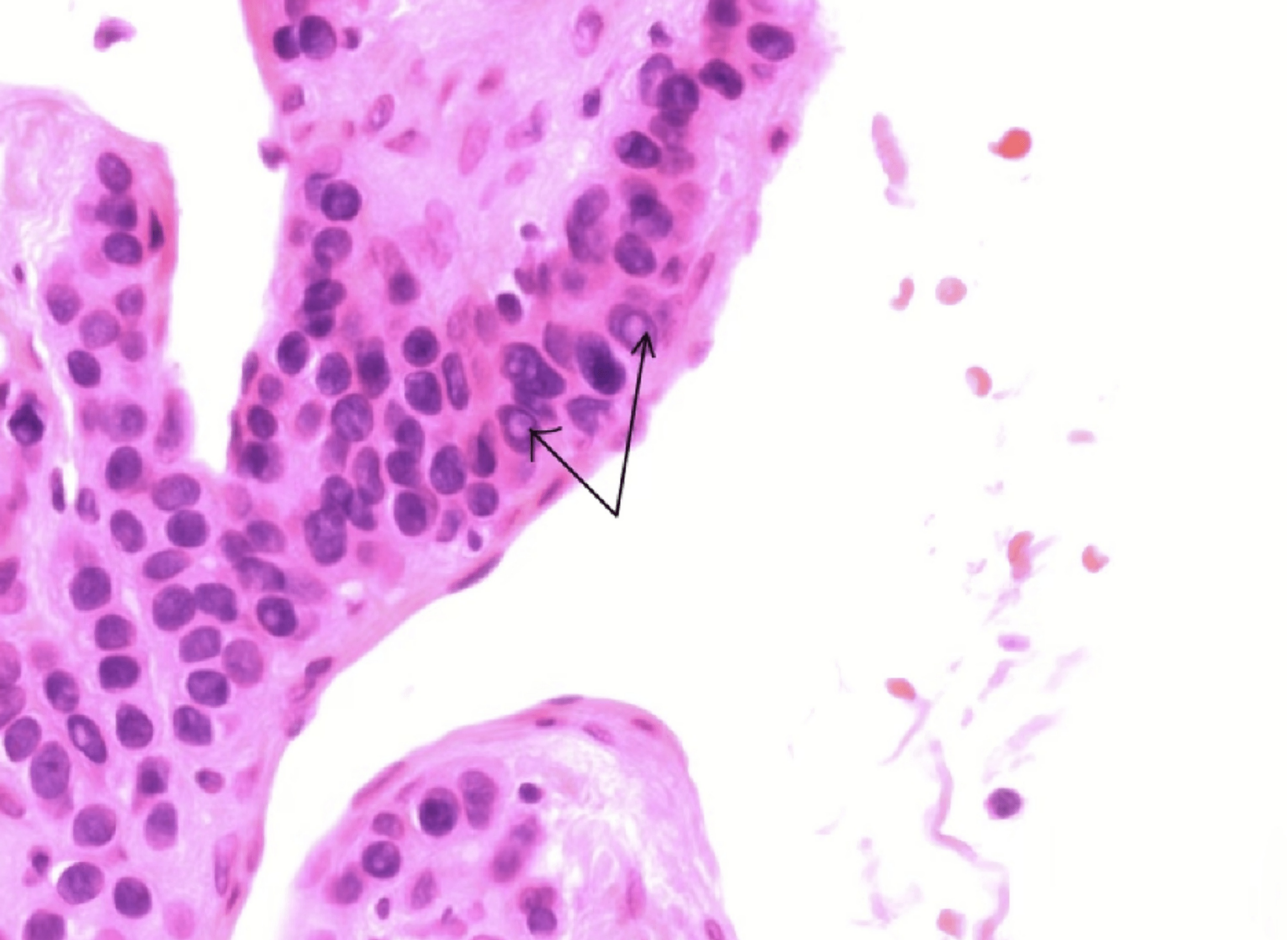
Intra-nuclear inclusion bodies of glomus tumor cells. A H&E stain under 600x magnification demonstrates intranuclear inclusions marked by the black arrows within the glomus cells, a feature occasionally observed in these tumors. The inclusions appear as eosinophilic or clear structures within the nuclei, causing nuclear enlargement and irregularity. These findings, along with the characteristic round glomus cells and perivascular arrangement, support the diagnosis of a glomangioma.

**Figure 2:**
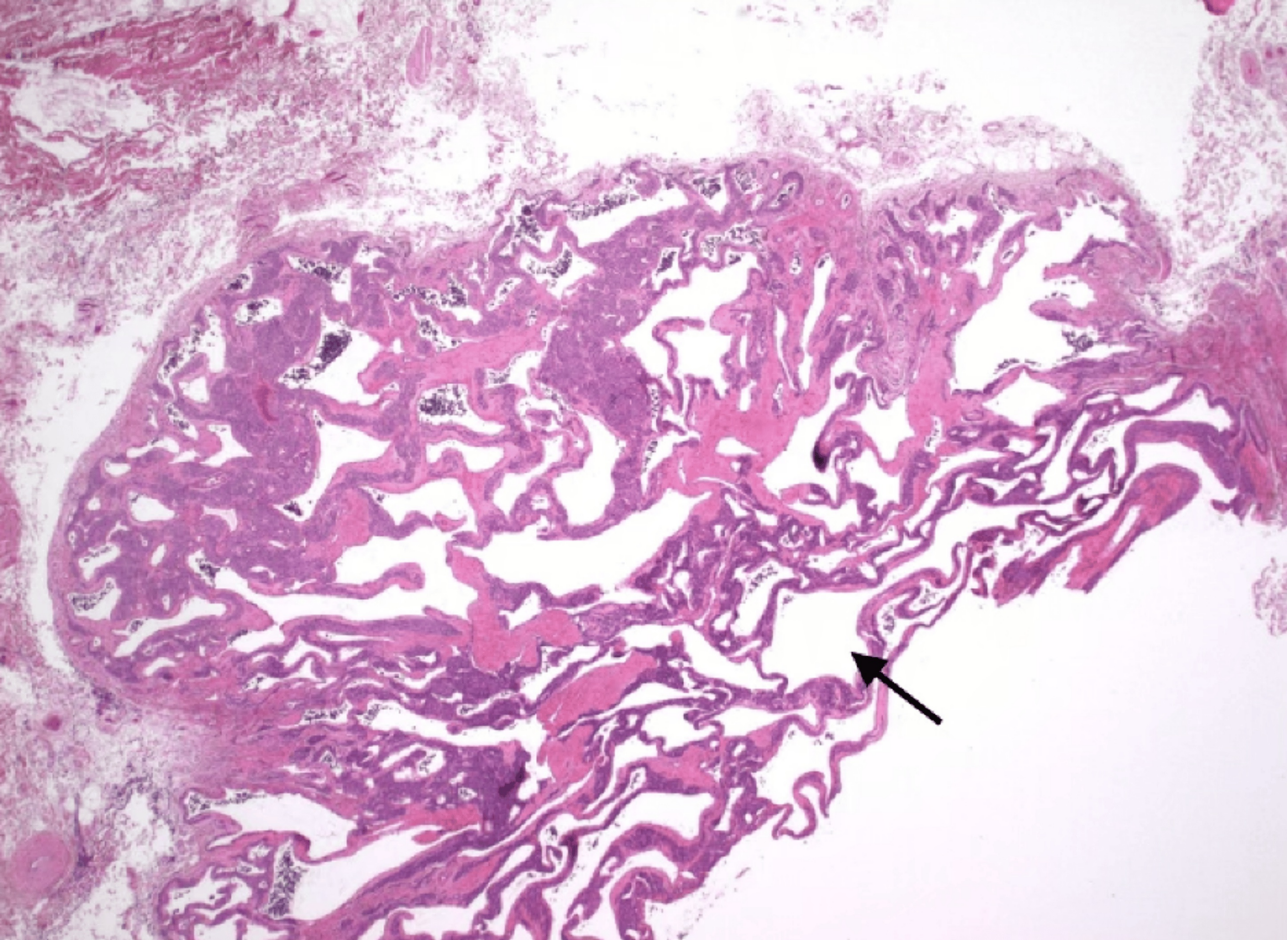
Histological stain of glomus tumor cells encasing microvasculature. This H&E stain under 20x magnification demonstrates vessels marked by the black arrows that are lined by endothelial cells and are encased by collars of uniform, round glomus cells with eosinophilic cytoplasm. The tumor exhibits a lobular architecture with branching vascular channels, a hallmark of glomangiomas.

**Figure 3:**
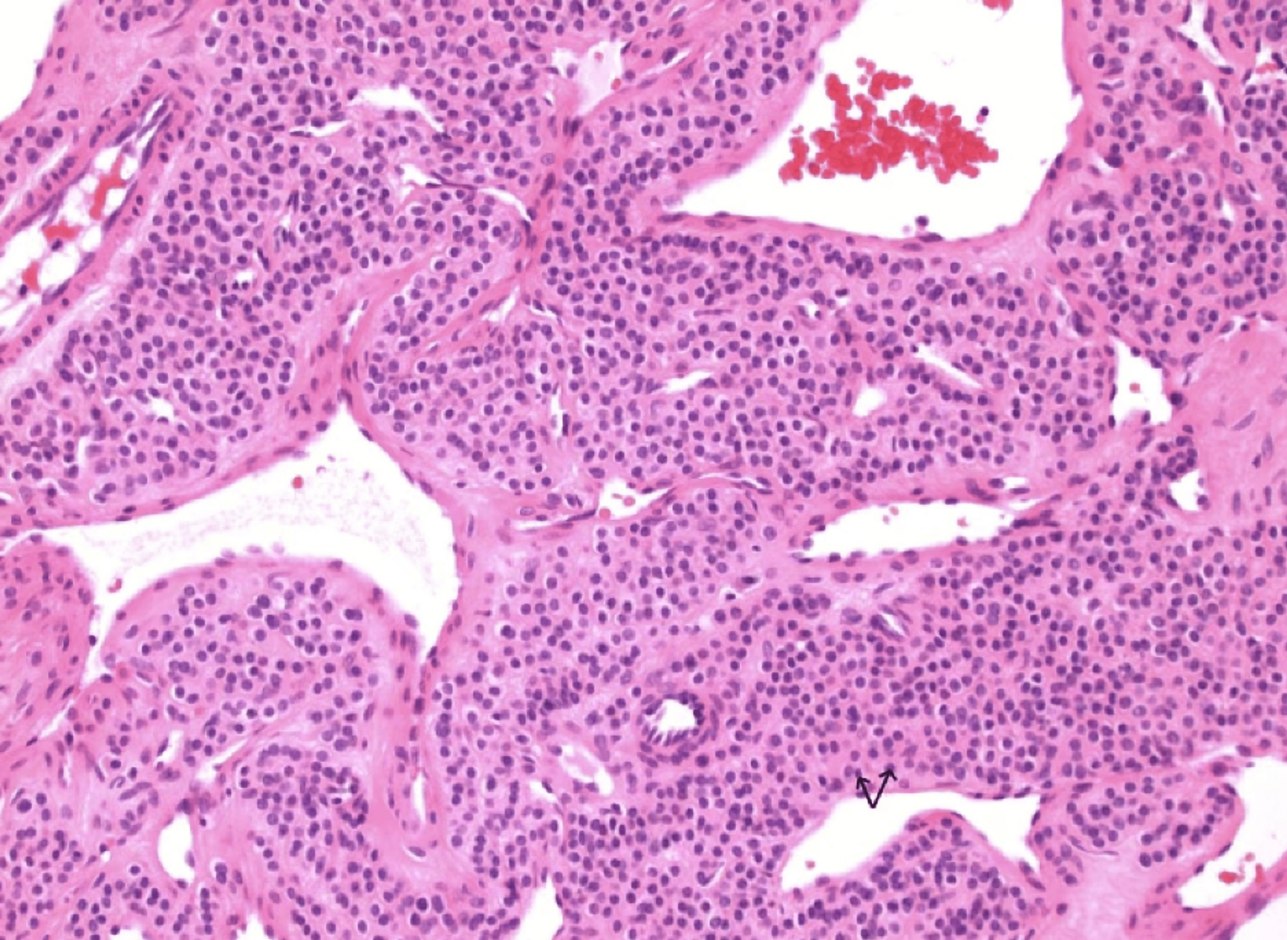
Histological architecture of glomus tumor. This H&E stain under 200x magnification demonstrates a dense proliferation of round glomus cells with scant eosinophilic cytoplasm and indistinct cell borders. The tumor cells are arranged in solid sheets surrounding dilated vascular spaces, which are lined by a thin endothelial layer. The overall architecture exhibits a well-organized lobular growth pattern, which is a recognized characteristic of glomangiomas.

Histological examination of the tissue samples using hematoxylin and eosin (H&E) staining revealed round to polygonal glomus cells with eosinophilic cytoplasm and centrally located basophilic nuclei with intranuclear inclusion bodies (Figure 1). These glomus cells were typically arranged around small, branching vascular channels, surrounded by flattened and elongated cells characteristic of endothelial cells (Figure 2). The tumor was often embedded within a myxoid or hyalinized stroma (Figure 3). Further analysis of the tumor cells displayed positivity for smooth muscle actin (SMA) and vimentin, reflecting the modified smooth muscle origin of the glomus cells. The histopathological analysis revealed features consistent with a glomangioma, including the characteristic arrangement of glomus cells around small vascular channels and immunohistochemical positivity for SMA and vimentin. The histological and surgical findings, including the lack of gross necrotic features and an absence of an infiltrative growth pattern, indicated no evidence of malignant potential. The patient was seen at follow-up and had no evidence of tumor reoccurrence or further complications.

## Discussion

The case presented here describes a glomangioma located near the right knee, an atypical and rare site for this type of lesion. The absence of the hallmark symptoms of pain or hypersensitivity and the lesion's mobile, asymptomatic nature initially led to a misdiagnosis of a sebaceous cyst, a common subcutaneous lesion type. This case exemplifies the diagnostic challenges posed by extradigital glomangiomas, which can mimic and can be mistaken for other more frequently encountered subcutaneous masses, including lipomas, epidermoid cysts, sebaceous cysts, or dermatofibromas. Although data on extradigital glomangiomas is limited, studies have reported that these tumors can occur in various parts of the body, including the trunk, extremities, head, and neck. A 20-year retrospective review of 50 glomus tumor cases found that 10% were located extra-digitally on the upper extremities [11]. The atypical presentation and location of our patient’s glomangioma underscore the importance of maintaining a broad differential diagnosis for persistent, unusual subcutaneous lesions.

Histopathological analysis remains the gold standard for diagnosing glomangiomas. In this case, the characteristic histopathological findings-round to polygonal glomus cells with eosinophilic cytoplasm, centrally located basophilic nuclei, and their arrangement around branching vascular channels-confirmed the diagnosis. Additionally, the tumor's immunohistochemical positivity for SMA and vimentin further corroborated its glomus cell origin. The SMA positivity reflects the smooth muscle lineage of glomus cells, while the vimentin positivity indicates their mesenchymal derivation [12,13]. These specific histopathological and immunohistochemical features are essential for the accurate identification and diagnosis of glomangiomas, particularly in atypical locations where the clinical presentation may not be typical.

The lack of hallmark symptoms in this case underscores the importance of maintaining a broad differential diagnosis for persistent subcutaneous masses, particularly those in atypical locations. Glomangiomas, while rare, should be considered when subcutaneous lesions are resistant to conventional treatments or fail to resolve after attempted drainage or aspiration. Surgical excision is both diagnostic and curative, as seen in this case, with histopathological examination serving as the definitive diagnostic tool. This approach is crucial for accurately identifying these lesions and providing timely, effective treatment, as glomangiomas can be easily mistaken for more common subcutaneous lesions. Early recognition and proper management are key to preventing recurrence and ensuring optimal patient outcomes.

Our case highlights the critical role of histopathological examination in accurately identifying extradigital glomangiomas and emphasizes the vital need for greater awareness among clinicians and pathologists regarding the potential for these rare tumors to present in atypical locations beyond the typical subungual or digital regions. Special care should be taken in the clinical setting, as the absence of classic symptoms does not preclude the presence of a glomangioma, particularly in extradigital locations. Clinicians must maintain a high index of suspicion and be aware that these rare tumors can present atypically, potentially leading to a misdiagnosis of more common osteomuscular conditions. A thorough clinical evaluation, coupled with appropriate diagnostic workup, is essential to correctly identify extradigital glomangiomas and avoid delayed or incorrect treatment [14,15]. Recognizing the diverse clinical presentations of glomangiomas is essential for providing timely and effective treatment, avoiding unnecessary interventions, and reducing the likelihood of recurrence. Enhanced familiarity with the diagnostic features and diverse manifestations of extradigital glomangiomas can significantly improve diagnostic accuracy, leading to more appropriate management strategies and improved outcomes for patients affected by these unusual lesions.

## Conclusions

Our case contributes to the limited body of literature on extradigital glomangiomas and underscores their potential to masquerade as more common subcutaneous lesions. A high index of suspicion, combined with histopathological confirmation, is essential for diagnosing and managing these rare tumors, especially in atypical presentations like the knee in our case. The findings presented in this case report further expand our understanding of the diverse clinical manifestations of glomangiomas, which can occur in locations beyond the typical subungual or digital regions. Recognizing the possibility of extradigital glomangiomas and maintaining a broad differential diagnosis when evaluating persistent subcutaneous lesions is crucial for ensuring timely and appropriate treatment and reducing the likelihood of recurrence. Our case highlights the importance of thorough clinical evaluation, targeted diagnostic workup, and close collaboration between clinicians and pathologists to accurately identify these uncommon but clinically significant lesions. By contributing this case to the growing body of literature, we aim to enhance awareness of extradigital glomangiomas and their unique clinical presentation in the knee region.
